# Exogenous oxygen is required for prostanoid induction under brain ischemia as evidence for a novel regulatory mechanism

**DOI:** 10.1016/j.jlr.2023.100452

**Published:** 2023-09-30

**Authors:** Drew R. Seeger, Brennon Schofield, Derek Besch, Svetlana A. Golovko, Peddanna Kotha, Meredith Parmer, Shahram Solaymani-Mohammadi, Mikhail Y. Golovko

**Affiliations:** Department of Biomedical Sciences, School of Medicine and Health Science, University of North Dakota, Grand Forks, ND, USA

**Keywords:** arachidonic acid, prostaglandins, cyclooxygenase, oxidized lipids, brain lipids, phopsholipids, head-focused microwave irradiation

## Abstract

Previously, we and others reported a rapid and dramatic increase in brain prostanoids (PG), including prostaglandins, prostacyclins, and thromboxanes, under ischemia that is traditionally explained through the activation of esterified arachidonic acid (20:4n6) release by phospholipases as a substrate for cyclooxygenases (COX). However, the availability of another required COX substrate, oxygen, has not been considered in this mechanism. To address this mechanism for PG upregulation through oxygen availability, we analyzed mouse brain PG, free 20:4n6, and oxygen levels at different time points after ischemic onset using head-focused microwave irradiation (MW) to inactivate enzymes in situ before craniotomy. The oxygen half-life in the ischemic brain was 5.32 ± 0.45 s and dropped to undetectable levels within 12 s of ischemia onset, while there were no significant free 20:4n6 or PG changes at 30 s of ischemia. Furthermore, there was no significant PG increase at 2 and 10 min after ischemia onset compared to basal levels, while free 20:4n6 was increased ∼50 and ∼100 fold, respectively. However, PG increased ∼30-fold when ischemia was followed by craniotomy of nonMW tissue that provided oxygen for active enzymes. Moreover, craniotomy performed under anoxic conditions without MW did not result in PG induction, while exposure of these brains to atmospheric oxygen significantly induced PG. Our results indicate, for the first time, that oxygen availability is another important regulatory factor for PG production under ischemia. Further studies are required to investigate the physiological role of COX/PG regulation through tissue oxygen concentration.

Prostanoids (PG), including prostaglandins, prostacyclins, and thromboxanes, regulate a variety of physiological and pathological processes in the central nervous system (CNS) such as sleep, neuroplasticity, angiogenesis, acute cerebral blood flow, inflammation, cancer, neurodegeneration, neuropsychiatric conditions, and CNS injury (1, 2, 3, 4, 5, 6, 7, 8, 9, 10, 11, 12, 13, 14, 15, 16). Knowing the dynamics and regulation of brain PG alterations is important for understanding the physiology and pathology of these processes as well as the development of therapeutic treatments for brain disorders.

Previously, we (17, 18, 19, 20, 21) and others (22, 23, 24, 25) have demonstrated a rapid (within seconds) and dramatic (∼30 fold) increase in brain PG upon global ischemia modeled by decapitation. Acute PG increase under these conditions is traditionally explained through the activation of arachidonic acid (20:4n6) cascade (16, 26, 27, 28, 29). In this pathway, the ischemia-associated depletion of tissue O_2_ and energy substrates leads to decrease in energy charge, which results in cytosolic calcium increase and activation of multiple calcium- and kinase-dependent lipases and consequent 20:4n-6 release. The rate-limiting enzymes for PG synthesis, cyclooxygenase 1 and 2 (COX), and downstream synthases convert released 20:4n-6 to PG, causing a dramatic PG increase upon ischemia. However, this traditional mechanism for PG regulation under ischemia and other conditions does not account for another critical substrate for COX activity, O_2_. A limited number of studies indicate that the Michaelis constant (K_m_) for COX by O_2_ (the O_2_ concentration at which COX activity is equal to half the maximal activity) is between 10 and 100 μM (30, 31), close to the brain tissue free O_2_ concentration of ∼50 μM as calculated from (32) after applying Henry’s Law. These data indicate that small alterations in tissue O_2_ concentrations might change PG production and may serve as another level of tissue COX activity regulation in addition to 20:4n6 release. Because O_2_ depletion may precede lipase activation upon brain ischemia, we speculated that PG are not increased under brain ischemia. Further, we proposed that the previously observed PG increase is associated with craniotomy required for brain removal for analysis when O_2_ becomes available for COX activity.

To test this mechanism, we analyzed mouse brain O_2_ concentrations, free 20:4n6, and PG levels at baseline, 30 s, 2, and 10 min after ischemia onset using head-focused microwave irradiation (MW) to inactivate enzymes in situ before craniotomy. We report a short (5.32 ± 0.45 s) O_2_ half-life [T_1/2_(O_2_)] in the ischemic brain, with O_2_ decreasing to undetectable levels (<10 nM) within 12 s of ischemia onset. During this time, we did not detect changes in free 20:4n6 or PG levels. At the increased duration of ischemia, free 20:4n6 levels were significantly (up to 100-fold) increased without significant PG increase compared to basal levels. However, PG increased ∼30-fold when ischemia was followed by craniotomy without MW, with a similar free 20:4n6 increase in both cases, indicating that 20:4n6 release is not the only factor required for 20:4n6 cascade activation. Moreover, craniotomy performed on nonMW mice under anoxic conditions did not result in PG induction in the ischemic brain, while exposure of these ischemic brains to atmospheric O_2_ dramatically induced PG, excluding tissue damage contribution to the PG increase in nonMW tissue, or PG degradation under MW.

Together with our and others’ previous reports on PG stability under MW conditions (18, 23), our results indicate, for the first time, that the ischemia event does not increase brain PG concentration, and PG production might be regulated through O_2_ availability. Further studies are required to validate the physiological and pathological role for COX activity regulation through tissue O_2_ concentration.

## Materials and methods

### Animals

This study was conducted in accordance with the National Institutes of Health Guidelines for the Care and Use of Laboratory Animals and under an animal protocol approved by the University of North Dakota IACUC (protocols 2103-5). Thirty-three male C57BL/6 mice at 4–6 months of age were used for experiments. Mice were given a standard laboratory chow and water ad libitum.

### Oxygen assay

**Fig. 1:**
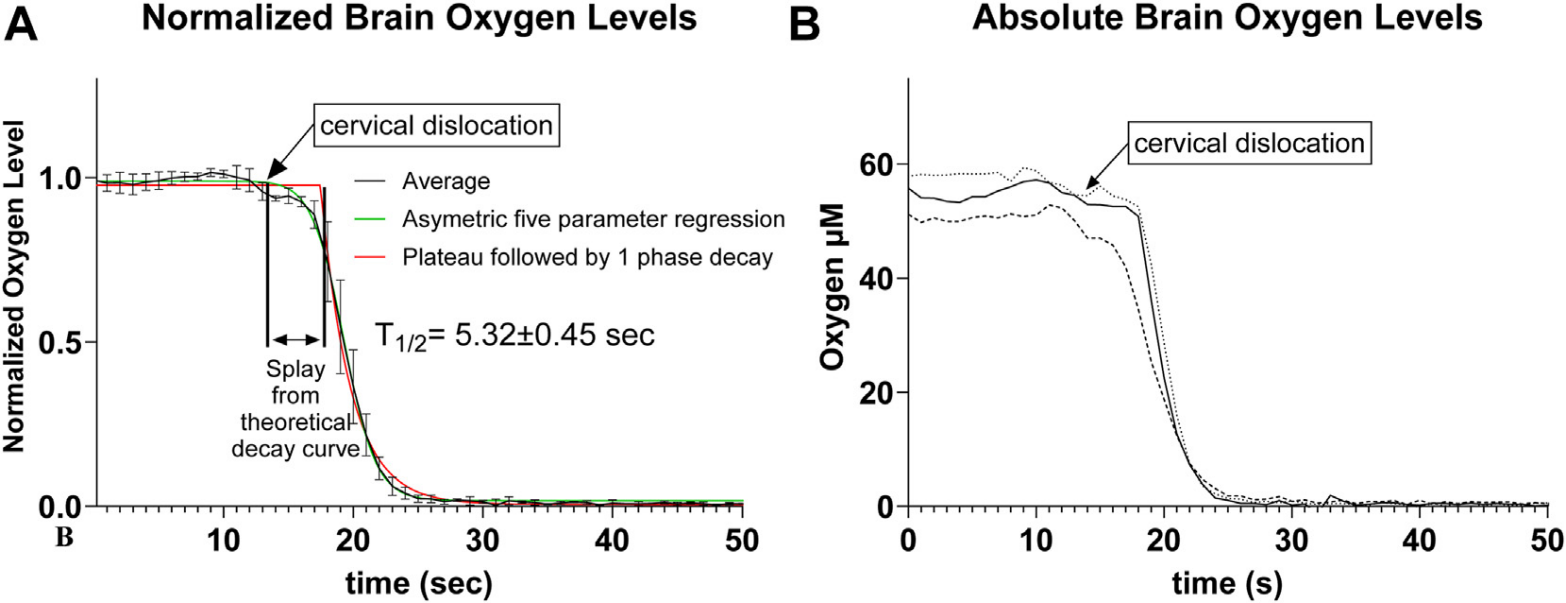
Oxygen level is rapidly depleted following global ischemia. Mice were fixed in a stereotaxic frame followed by oxygen microsensor insertion into cortex. Global ischemia was induced by cervical dislocation. A: Normalized cortex oxygen levels used to calculate O_2_ half-life (T_1/2_). Values are mean ± standard deviation (n, number of animals, =3) with the individual absolute value presented in panel B. T_1/2_ was calculated using nonlinear regression in GraphPad Prism 10 as described in the Materials and Methods. B: Absolute levels of cortex O_2_ before (54.6 ± 3.2 μM) and during global ischemia.

To determine O_2_ concentration, an O_2_ microsensor probe (Unisense OX-10 microsensor, Aarhus Denmark) was inserted into the mouse cortex. Mice were anesthetized with ketamine (100 mg/kg) (Dechra Pharmaceuticals, Northwich, United Kingdom) and xylazine (10 mg/kg) (Covetrus, Portland, ME) and mounted in a temperature controlled (Kent Scientific, Torrington, CT) stereotaxic surgery device (Stoelting, Wood Dale, IL) to maintain mouse body temperature at 37°C using a rectal probe. A 1 × 1 mm borehole in the cranium was used to insert a calibrated 10-μm tipped O_2_ microsensor probe (Unisense OX-10 microsensor, Aarhus, Denmark) to record the cortex O_2_ concentration, −1 mm DV, 2 mm ML, and −2 mm AP to bregma (33). Bregma and lambda were aligned as described in (33). Because the small (10 μm) probe diameter did not leave a visible trace in the brain sections, we were unable to directly confirm the probe position in the cortex. Thus, we inserted a pulled glass capillary filled with Evans blue dye at the same coordinates to visualize probe placement in a separate group of mice (supplemental Fig. S1). O_2_ levels were allowed to stabilize for 10–20 min followed by cervical dislocation. Data were compiled and T_1/2_(O_2_) was calculated using statistical software (GraphPad Prism 10, Boston, MA). Specifically, an asymmetric five-parameter regression analysis gave the best fit to the O_2_ concentration dynamics and was used to calculate observed T_1/2_(O_2_), while plateau followed by 1-phase decay regression analysis was used to calculate a splay from theoretical decay curve on Fig. 1A. A separate group of mice was used to collect brain tissue for PG analysis.

### Mouse brain collection from ischemic and control groups under atmospheric O_2_

For non-microwave irradiated (nonMW) ischemic animals (nonMW group), mice were anesthetized with isoflurane and euthanized by cervical dislocation. Brains were removed at 0.5, 2, and 10 min postmortem to model the corresponding durations of global brain ischemia, and then immediately frozen in liquid N_2_. Mice from the 2- and 10-min ischemia groups were placed on a 37°C heating mat before brain collection to maintain post-mortem enzymatic activity. Brain removal from the cranial vault took 30 s. This brain collection method was commonly used in previous studies (17, 22, 34) and exposes metabolically active brain tissue to high atmospheric O_2_ concentrations before tissue analysis. Another group of mice was exposed to microwave irradiation (MW) to denature enzymes in situ at 0 s (control group), 30 s, 2, and 10 min after cervical dislocation (ischemic MW group). Similar to the nonMW group, mice from the 2- and 10-min ischemia groups were placed on a 37°C heating mat. The brains from the MW group were metabolically inactive when exposed to high O_2_ levels before tissue analysis. Whole frozen brains were pulverized into a homogenous powder under liquid N_2_ before analysis.

### Mouse brain collection under anoxic conditions

Mice were anesthetized with isoflurane, euthanized by cervical dislocation, and immediately placed into a Bactrox hypoxic chamber (Sheldon Manufacturing, Cornelius, OR) flushed with N_2_ gas to undetectable O_2_ concentrations (<0.1% O_2_) measured by a Cytocentric O_2_ controller (BioSpherix, Parish, NY). The whole brain was collected at 30 s after cervical dislocation as described above, divided into two hemispheres. One brain hemisphere was pulverized into a homogenous powder under liquid N_2_ and extracted inside the anoxic chamber, while another was processed under normal atmospheric O_2_.

### Analysis of free arachidonic acid

Brain tissue powder (∼10 mg) was weighed and sonicated in 90 μl of methanol containing 0.02% BHT (35, 36) and 200 ng of arachidonic acid-d_8_ (Cayman Chemical, Ann Arbor, MI) as an internal standard. Following centrifugation (12,000 *g* 15 min), a 10 μl aliquot of supernatant was dried under N_2_ gas, redissolved in 1 ml acetonitrile:2-propanol:water (1:1.28:1.28 by volume), and 10 μl was injected into the ultra-high-pressure liquid chromatography - mass spectrometry (UPLC-MS) system for analysis as previously described (35, 36).

The UPLC system consisted of a Waters ACQUITY UPLC pump with a well-plate autosampler (Waters, Milford, MA) equipped with an ACUITY UPLC HSS T3 column (1.8 μm, 100 Å pore diameter, 2.1 × 150 mm; Waters) and an ACUITY UPLC HSS T3 Vanguard precolumn (1.8 μm, 100 Å pore diameter, 2.1 × 5 mm; Waters). The column temperature was set at 55°C and the autosampler temperature was set at 8°C. LC solvent A consisted of acetonitrile:water (40:60) with 10 μM ammonium acetate and 0.025% acetic acid. Solvent B was acetonitrile:2-propanol (10:90) containing 10 μM ammonium acetate and 0.02% acetic acid. The flow rate was 0.3 ml/min for the duration of the run and the initial solvent B was 30%. At 0.1 min, %B was increased to 54% over 10 min, and then to 99% B over 10 min. 99% B was held for 8 min and then returned to initial conditions over 0.5 min. The column was equilibrated for 2.5 min between injections.

Free 20:4n6 was quantified using a quadrupole time-of-flight mass spectrometer (Q-TOF, Synapt XS, Waters) with electrospray ionization in negative mode. 20:4n6 was monitored in the low energy channel at 303.2324 m/z and 20:4n6-d_8_ at 311.2826 m/z with a mass window of 0.02. Lock spray for mass correction (leucine enkephalin, 100 pg/μl) was infused at a rate of 10 μl/min. 20:4n-6 was confirmed by coelution and quantified using a standard curve built against 20:4n6-d_8_ internal standard (Cayman Chemical Co). MassLynx V4.1 software (Waters) was used for instrument control, acquisition, and sample analysis. Free 20:4n6 values are expressed as μmol per kg wet tissue weight (μmol/kgww).

### PG analysis

Following pulverization, PG was extracted using a liquid/liquid extraction method as previously described (18). Pulverized brain tissue (∼20 mg) was weighed into a Tenbroeck homogenizer containing 3 ml of 2:1 acetone:saline with PGE_2_d_9_ (Cayman Chemical Co) as an internal standard. Samples were homogenized and transferred to silanized (Sigmacote, Sigma, St. Louis, MO) screw top tubes, and subjected to centrifugation to remove proteins. Following centrifugation, supernatants were transferred to a new silanized screw top tube. Extracts were washed three times with 2 ml of *n*-hexane to remove non-polar compounds, 20 μl of 2 M formic acid was added for acidification, and 2 ml of chloroform containing 0.005% BHT was added to extract PG. Chloroform extracts were concentrated under a stream of N_2_, transferred to silanized microinserts (MicroSolv, Leland, NC), dried under N_2_, and redissolved in 25 μl of 1:1 acetonitrile:water. Ten μl was injected onto UPLC-MS for analysis. UPLC-MS/MS analysis was performed on a Waters TQS MS in multiple reaction monitoring (MRM) mode with electrospray ionization operated in negative ion mode as previously described (19, 37).

The UPLC system consisted of a Waters ACQUITY UPLC pump with a well-plate autosampler (Waters) equipped with an ACUITY UPLC HSS T3 column (1.8 μm, 100 Å pore diameter, 2.1 × 150 mm; Waters) and an ACUITY UPLC HSS T3 Vanguard precolumn (1.8 μm, 100 Å pore diameter, 2.1 × 5 mm; Waters). The column temperature was set at 55°C and the autosampler temperature was set at 8°C. LC solvent A consisted of water with 0.1% formic acid and solvent B was acetonitrile with 0.1% formic acid with a flow rate of 0.45 ml/min. Initially, solvent B was held at 39% for 0.5 min, then increased to 40.5% over the next 6.88 min, and finally increased to 98% over 0.2 min 98% B was held for 7 min and returned to initial over 0.2 min. The column was equilibrated for 2 min between injections.

PG were quantified using PGE_2_d_9_ as an internal standard that has been previously validated as an internal standard for all prostanoids analyzed (18). PG were monitored in MRM mode with the following mass transitions as previously described (37): PGE_2_ - 351.18/271.13; PGD_2_ - 351.06/271.14; 6-ketoPGF_1α_ - 369.26/163.07; PGF_2α_ - 353.07/193.04; TXB_2_ - 369.20/169.00. MassLynx V4.1 software (Waters) was used for instrument control, acquisition, and sample analysis. PG values are expressed as ng per g wet tissue weight (ng/gww).

### Statistical analysis

Statistical analysis was performed using GraphPad Prism 10 (GraphPad, Boston, MA). Statistical significance was determined by one-way ANOVA with the Tukey *post hoc* test. Values were considered significant with *P* value <0.05 and are expressed as mean ± SD. T_1/2_(O_2_) was calculated using nonlinear regression in GraphPad Prism 10 as described above.

## Results

### Cortical oxygen levels during global ischemia

To quantify brain O_2_ during ischemia, we used a microsensor O_2_ probe (Unisense OX-10) inserted into the cortex. The small, 10 μm diameter tip of this probe allows for a real-time, tissue-specific free O_2_ level measurement in the living animal with minimal mechanical damage and hypoperfusion due to reduced vascular compensation, and minimal O_2_ consumption by the probe. Free O_2_ levels were rapidly (within 12 s) depleted after the ischemia onset with T_1/2_(O_2_) of 5.32 ± 0.45 s (Fig. 1A). There was a splay from the theoretical exponential decay curve (4 s, Fig. 1A) that might be explained by the diffusion latency of O_2_ between the extracellular fluid where O_2_ was measured, and the cellular fluid where O_2_ was fast consumed upon ischemia. Thus, it is possible that the cellular T_1/2_(O_2_) is shorter than the reported values. Consistent with previous reports (38, 39), the basal cortical O_2_ levels were 54.6 ± 3.2 μM (Fig. 1B). These data indicate that O_2_ levels fall below *Km(O*_*2*_*)* values of COX1/2 (in the 10 μM range (30, 31, 40, 41)) within seconds of global ischemia onset.

### PG production during ischemia

**Fig. 2:**
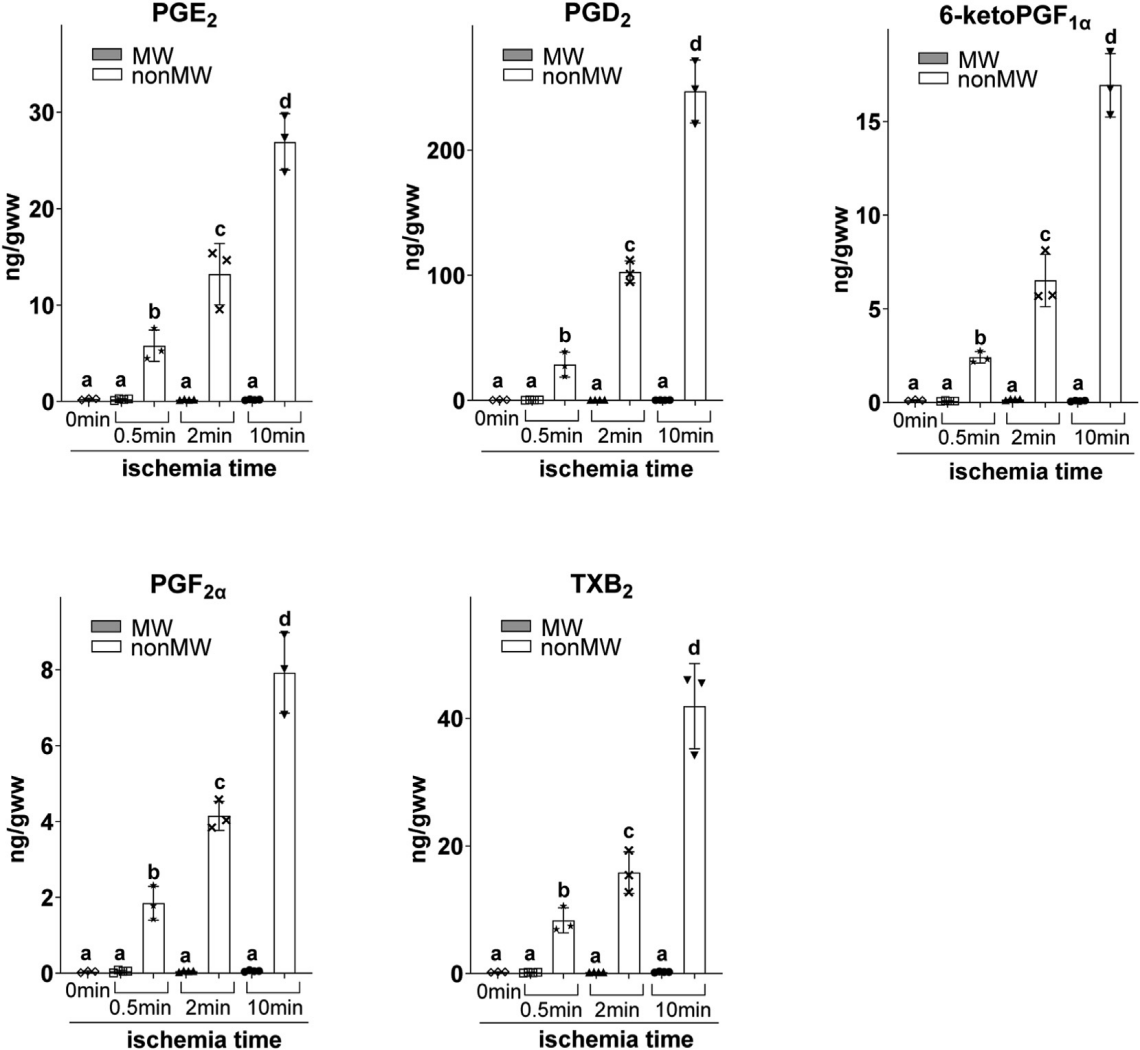
Prostanoids are not produced in the ischemic brain. Global brain ischemia was modeled by cervical dislocation. After cervical dislocation, brains were removed from the cranial vault either after microwave irradiation to heat denature enzymes in situ (MW) or collected without microwave irradiation (nonMW) at 0.5, 2, and 10 min postmortem. The MW group represents true PG levels in the ischemic and control brains without the artificial contribution of exogenous atmospheric O_2_ during tissue handling, while the nonMW group represents PG levels affected by atmospheric O_2_ during tissue handling. Postmortem mouse body temperature was maintained at 37°C by wrapping mice with a temperature-controlled heating pad to maintain enzymatic activity following cervical dislocation. Basal prostanoids (PG) levels were determined in MW brain tissue without cervical dislocation (0 min of global ischemia). PG were quantified by UPLC-MS against stable isotope labeled internal standard. Values are mean ± standard deviation (n = 3–4) with individual values. Values that do not share the same letter are statistically different (*P* ˂ 0.05, one-way ANOVA with Tukey’s *post hoc* test).

Next, we determined if previously reported rapid PG production following global brain ischemia (17, 18, 19, 20, 21, 22, 23, 24, 25) is associated with metabolically active tissue exposure to atmospheric O_2_ during tissue removal from the cranium, rather than production in situ in the ischemic brain. In these experiments, we measured brain PG production at increasing ischemia durations followed by MW fixation to heat denature enzymes in situ (true PG levels without exogenous atmospheric O_2_ contribution to ischemic brain metabolome) and without MW (exogenous atmospheric O_2_ contribution to PG levels). Following global ischemia, we did not detect alterations in PG levels after in situ enzyme inactivation by MW (MW group) when compared to basal brain PG levels (MW, 0 min ischemia) (Fig. 2). However, at all time points after global ischemia, PG were significantly increased when metabolically active brains were exposed to atmospheric O_2_ by removal from the cranium without MW (nonMW group, Fig. 2). Compared to the MW group, PGE_2_ levels in nonMW brain tissue were increased 23-, 53-, and 109-fold at 0.5, 2, and 10 min of global ischemia, respectively. Similarly, we did not detect alterations in PGD_2_, 6-ketoPGF_1α_, PGF_2α_, and TXB_2_ levels after MW when compared to basal brain PG levels (Fig. 2). Likewise, PGD_2_ (63-, 226-, 544-fold), 6-ketoPGF_1α_ (20-, 55-, 143-fold), PGF_2α_ (46-, 104-, 198-fold), and TXB_2_ (36-, 69-, 182-fold) levels at 0.5, 2, and 10 min, respectively, are all significantly increased following exposure to atmospheric O_2_ without MW (Fig. 2). These data indicate that PG are not produced upon global brain ischemia as previously reported, but are instead produced when enzymatically active brain tissue is exposed to exogenous O_2_.

### Non-esterified 20:4n6 levels during global ischemia

**Fig. 3:**
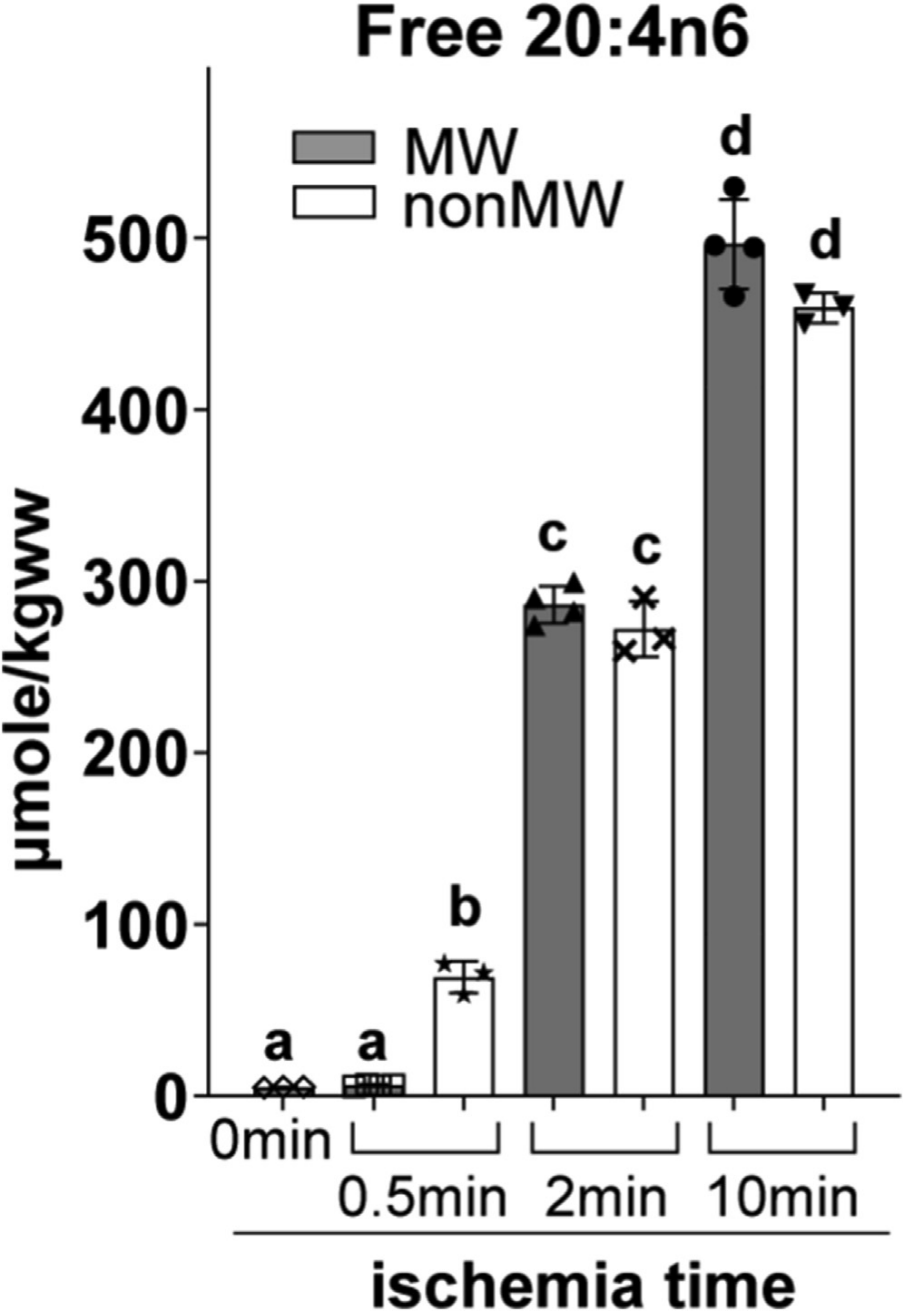
Brain free arachidonic acid (20:4n6) levels are unaffected by microwave irradiation during global ischemia. Mice were either microwave irradiated to inactivate enzymes (MW), or collected without microwave irradiation (nonMW) at 0.5, 2, and 10 min postmortem. Basal free 20:4n6 levels were determined in MW brain tissue at 0 min of global ischemia. Free 20:4n6 levels were determined by UPLC-MS. Values are mean ± standard deviation (n = 3–4) with individual values. Values that do not share the same letter are statistically different (*P* ˂ 0.05, one-way ANOVA with Tukey’s *post hoc* test).

Because 20:4n6 is an essential substrate for COX, and 20:4n6 availability is considered to be the major regulatory mechanism for PG production under different conditions, including brain ischemia (16, 26, 27, 28, 29), we determined free 20:4n6 dynamics during brain ischemia, as well as the effect of MW on 20:4n6 levels at basal and ischemic conditions. MW did not affect free 20:4n-6 levels at 2 and 10 min when compared to the nonMW time counterpart (Fig. 3), which did not correlate with PG dynamics (Fig. 2). However, at 0.5 min, 20:4n6 level in nonMW brain was significantly increased compared to basal and MW 0.5 min 20:4n6 levels (13- and 11-fold, respectively). We speculate that this increase in free 20:4n6 is a result of mechanical damage during craniotomy and not due to phospholipase activity during extraction, or 20:4n6 degradation under MW conditions, as 20:4n6 levels in nonMW and MW at 2 and 10 min of ischemia were not significantly different from their time point counterpart. Consistent with this speculation, previous research shows that traumatic injury increases PLA_2_-mediated 20:4n6 release in the brain (42, 43). Importantly, while MW and nonMW 20:4n6 levels are unchanged at 2 and 10 min of ischemia compared to their respective time point, PG production is significantly increased in nonMW brains (Fig. 2). This suggests that while free 20:4n6 levels are increased during global ischemia, availability of O_2_ is crucial for PG production. Thus, the rapid depletion of free O_2_ in the ischemic brain precedes 20:4n6 release and therefore PG production does not proceed in the intact cranial vault.

### PG are not increased in the ischemic nonMW brains handled under anoxic conditions

**Fig. 4:**
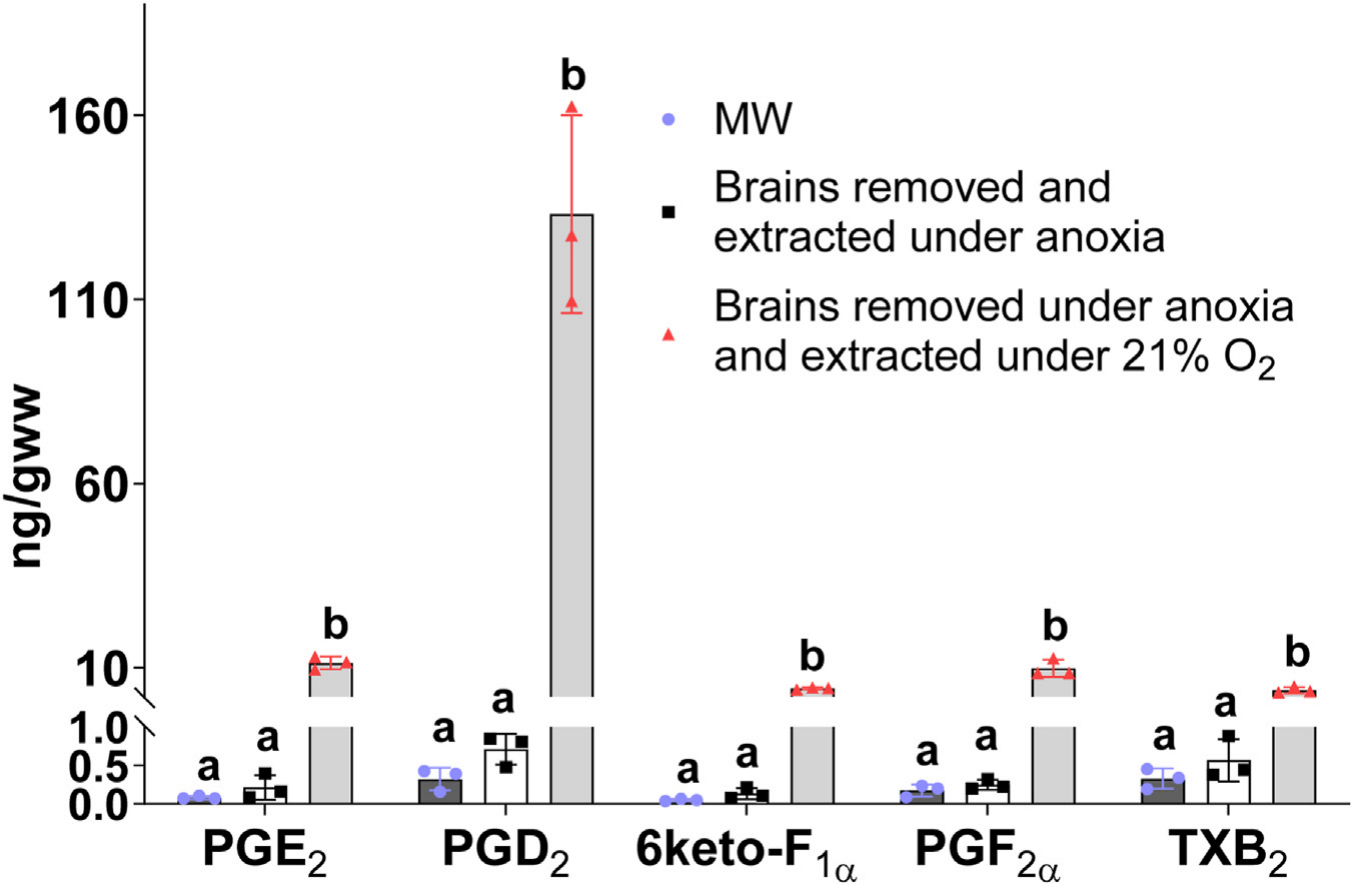
Prostanoids are not increased in the ischemic nonMW brains handled under anoxic conditions. Mice were subjected to 0.5 min global ischemia by cervical dislocation and brains were collected either after microwave irradiation to quench postmortem metabolism (MW, black bars) or collected without MW (nonMW) under anoxia in the anoxic chamber. After freezing in liquid nitrogen, one hemisphere was pulverized to a homogenous powder, and extracted in the anoxic chamber (white bars), while another hemisphere from each mouse was removed from the anoxic chamber and processed under atmospheric O_2_ (gray bars). Prostanoid levels were determined by UPLC-MS. Values mean ± standard deviation (n = 3–4) with individual values. Values that do not share the same letter are statistically different (*P* ˂ 0.05, one-way ANOVA with Tukey’s *post hoc* test).

To further confirm that exogenous atmospheric O_2_ rather than ischemia or brain injury during craniotomy contributes to PG induction, and to bolster findings that MW does not destroy endogenous PG that are potentially produced during ischemia, we collected ischemic (0.5 min) nonMW brains under an anoxic environment. There was no difference in PG levels between MW tissue and nonMW tissue removed and extracted under anoxia (Fig. 4), suggesting that prostanoids are not induced in these injured ischemic samples, and MW does not destroy endogenous PG. Interestingly, when nonMW brains are collected under anoxic conditions but pulverized and extracted under atmospheric O_2_, there is a robust and significant increase in PG (Fig. 4). These data suggest that increased PG in nonMW ischemic brain samples (Fig. 2) is likely a result of PG production during sample preparation due to exposure to atmospheric O_2_, but not the result of brain injury per se or an artifact of MW. Thus, O_2_ availability at any step during nonMW sample handling post-craniotomy increases PG levels.

## Discussion

In the present study, we addressed, for the first time, a novel mechanism for PG upregulation through O_2_ availability. We report that none of the measured PG are increased in the ischemic brain, and the previously reported rapid and dramatic PG upregulation upon brain ischemia (17, 18, 19, 20, 21, 22, 23, 24, 25) is the result of brain tissue exposure to atmospheric O_2_ during tissue removal from the skull. Considering the relatively low affinity of COX to O_2_, the described mechanism for oxygen-dependent PG production might have a significant role in PG regulation under other pathological or physiological conditions.

Previously, one study (23) demonstrated a significant 8-fold increase in TXB_2_ concentration in the MW ischemic brain with little endogenous PGE_2_/D_2_ induction, while other PG were not assayed in this study. The difference between TXB_2_ and PGD_2_ formation was explained through a different coupling between COX and downstream PG synthases. In the present study, we demonstrated, for the first time, no increase in any of 5 major COX-dependent products of 20:4n6 metabolism under brain ischemia including TXB_2_, and an O_2_-dependent mechanism for PG formation in the brain. To the best of our knowledge, no previous studies have addressed the relation between O_2_ concentration and PG production in the ischemic brain. It is difficult to speculate regarding previously reported TXB_2_ increase in the ischemic intact brain (23), a finding which was not reproduced in the present study. It is possible that differences in sample handling or processing might contribute to the discrepancy in the results. For example, the decapitation used by (23) allows the injured tissue to be exposed to O_2_, thus maintaining some COX activity, while cervical dislocation used in the present study prevents this artifact.

Traditionally, a rapid PG increase upon ischemic onset is explained through an activation of 20:4n6 cascade (16, 26, 27, 28, 29). In this pathway, an ischemia event leads to tissue energy charge decrease, thus leading to the suppression of ATP-dependent ion pumps and consequent cytosolic Ca^2+^ increase with activation of Ca- and kinase-dependent phospholipases. Activated phospholipases release 20:4n6 as a substrate for COX. The product of COX activity, PGG_2_ is then converted to PGH_2_ and later, to distinct end-products of PG by downstream enzymes.

However, this traditional view on PG upregulation does not consider the second essential COX substrate, O_2_, as two molecules of O_2_ are required for the cyclooxygenase reaction (44, 45). It is well established that in the ischemic brain, O_2_ decrease leads to energy deprivation and suppression of ATP-dependent ion pumps. Thus, O_2_ decrease precedes ion disbalance, including cytosolic Ca^2+^ increase (46), and might precede Ca- and kinase-dependent PLA_2_ activation required for 20:4n6 release for PG synthesis. To this end, it is important to establish the exact dynamics of brain O_2_ concentration during ischemia and estimate if COX is still active under O_2_ concentrations in the ischemic brain when 20:4n6 is released.

In order to accurately measure mouse cortex O_2_ concentration alteration under global ischemia in the present study, we used a small, 10 μm diameter O_2_ electrode to prevent tissue damage and possible hypoperfusion from surrounding tissue compression and to minimize O_2_ consumption by the electrode. Our data indicate a rapid decrease in O_2_ from 54 ± 3 μM to undetectable levels (<10 nM) within 12 s of ischemia onset with the calculated T_1/2_(O_2_) 5.32 ± 0.45 s (Fig. 1). However, it is possible that the cellular T_1/2_(O_2_) is even shorter than the reported values because we observed a 4 s splay from theoretical exponential decay curve (Fig. 1A). The splay might be the result of diffusion latency of O_2_ from the extracellular fluid where O_2_ was measured, to the cellular fluid where O_2_ was fast consumed upon ischemia. Consistent with the expected delay in PLA_2_ activation discussed above, we did not detect free 20:4n6 changes after the first 30 s of ischemia (Fig. 3). At 2 and 10 min of ischemia, we detected a significant (over 100-fold) increase in free 20:4n6 (Fig. 3) without any significant increase in PG (Fig. 2), which is consistent with the absence of PG synthesis in the ischemic brain when O_2_ concentration falls significantly below *Km(O*_*2*_*)* values in the 5–10 μM range. In addition, metabolically active tissue harvested and processed under anoxic conditions did not increase PG levels, while the same tissue exposed to atmospheric O_2_ dramatically increased all measured PG (Fig. 4). These data indicate, for the first time, that O_2_ depletion precedes 20:4n6 release upon brain ischemia, and PG are not produced in the ischemic brain. This mechanism does not exclude the role for 20:4n6 release in PG regulation in the presence of sufficient O_2_ concentrations. In this regard, liberated free 20:4n6 is readily available for PG formation in the ischemic tissue when O_2_ concentration raises above *Km(O*_*2*_*)*, and explains a burst PG induction in the ischemic brain upon exposure to atmospheric O_2_ (Figs. 2, 4). Our data indicate that O2 availability is an additional mechanism to regulate PG production that still requires 20:6n6 liberation.

Importantly, a few previous studies established a relatively low affinity of COX enzymes to O_2_. Depending upon the enzyme source and assay protocol, *Km(O*_*2*_*)* value for COX-2 ranges between 13 μM for mouse (40) and 10–16 μM for human (30, 31, 41) enzymes when 20:4n6 was used as a substrate. Similarly, low affinity was shown for COX-1 with *Km(O*_*2*_*)* ranging from 5.5 μM for ram (47) to 9–10 μM for human (31, 41) and sheep (40) enzymes. Low affinity to O_2_ highlighted by *Km(O*_*2*_*)* values indicate that COX activity will be significantly decreased when tissue O_2_ concentration decreases below 5–10 μM while the electron transport chain required for ATP production is still functional as *Km(O*_*2*_*)* for these oxygenases are below 1 μM (48, 49). These previously reported data are consistent with the absence of 20:4n6 release during first 30 s of ischemia, and the absence of PG synthesis under low O_2_ in the ischemic brain observed in the present study.

However, a number of artifacts and alternative mechanisms might contribute to the absence of PG upregulation in the ischemic tissue reported in the present study. First, for the samples where no PG upregulation was detected, we used MW to heat denature enzymes for PG synthesis in situ before tissue exposure to atmospheric O_2_ (Fig. 2). Therefore, it is reasonable to consider that PG, that are known for their instability, were degraded or trapped in denatured protein upon MW, thus contributing to the lower levels observed in MW tissue. We and others have previously addressed this concern by measuring recovery of the endogenously induced PG after MW (18), or the recovery of exogenous labeled PG injected into tissue before and after exposure to MW (23, 24). These studies clearly demonstrated that MW does not alter tissue PG levels and this is a safe “gold standard” method to prevent PG alterations during analysis.

Another consideration is the contribution of tissue injury to the observed difference in PG levels between ischemic MW and ischemic nonMW brains. MW samples were fixed before brain removal from the cranium, thus no injury was induced in these brains before the de-activation of enzymes. However, in nonMW ischemic samples, enzymes were active during brain removal from the cranium, causing injury-like damage which might account for additional PG upregulation in the ischemic samples. To test this mechanism, we analyzed nonMW brains removed from the cranium and extracted under anoxic conditions in a glove box. We did not detect PG upregulation in these “injured” samples (Fig. 4). However, when the same samples were exposed to atmospheric O_2_ during sample pulverization and extraction, PG were significantly upregulated (Fig. 4). Importantly, PG are actively synthesized during sample preparation and extraction procedures when the tissue powder is exposed to atmospheric O_2_ (18), thus PG are further upregulated during sample preparation and PG upregulation in nonMW tissue does not require instantaneous O_2_ diffusion into the brain tissue during removal from the cranium. Together, these data confirm that O_2_ availability but not tissue injury on its own is responsible for PG upregulation in nonMW brains and that PG are not upregulated in the ischemic brain.

In addition, we cannot exclude the effect of anesthesia on PG metabolism. In the present study, we used ketamine/xylazine for O_2_ sensor insertion since, in contrast to isoflurane, it does not require inhalation of O_2_ to keep animals under anesthesia during the surgery, eliminating a possible artifact from brain hyperoxia when isoflurane is used to measure brain O_2_ dynamics. However, we used isoflurane to briefly anesthetize mice before cervical dislocation and subjected them to MW to be consistent with our previous reports on brain PG metabolism (15, 19, 21). To address a possible effect of anesthesia on brain T_1/2_(O_2_), O_2_ dynamics were measured under both anesthetics upon ischemia. Under isoflurane, brain T_1/2_(O_2_) was 3.58 ± 1.47, not significantly different from ketamine/xylazine (5.32 ± 0.45 s, Fig. 1), indicating that the form of anesthesia does not significantly change brain O_2_ dynamics under ischemia. Importantly, anesthesia also affects brain fatty acid metabolism under basal and ischemia conditions. Deep pentobarbital anesthesia reduces palmitate turnover in brain phospholipids (50), and attenuates 20:4n6 liberation during global ischemia (51), though the effects of isoflurane or ketamine anesthesia have not been reported. It is reasonable to expect that anesthesia also affects PG production which depends upon 20:4n6 liberation. However, because all control and experimental animal groups used for PG analysis were subjected to the same anesthesia conditions, it is unlikely that anesthesia contributed to the observed differences in PG alterations between MW, nonMW, and N_2_-exposed brains.

Our reported findings might have an implication for other models and conditions such as injury and neuroinflammation. There is increasing evidence that PG-induced neuroinflammation contributes to clinical outcomes in traumatic brain injury (TBI), as reviewed in (52). Our data suggest that confounding differences in TBI models (closed vs. open head injury) could be exacerbated by varying levels of O_2_ availability during trauma, contributing an additional level of PG regulation that might also be applied to clinical outcomes of human TBI or brain surgery. Additionally, our results indicate that the depth of O_2_ penetration into the injured tissue, as well as the degree of tissue oxygenation before the injury, might be related to the difference in oxylipin production in different brain regions, with the highest level in the cortex and lowest in the brainstem (53).

Importantly, brain O_2_ concentrations in some brain regions are within the COX *Km(O*_*2*_*)* values and range from 1 μM O_2_ in pons to 55 μM in cortex to 88 μM in pia in rats (38), with similar values in cats and rabbits (54), and 60 μM in the mouse somatosensory cortex (39). Therefore, it is reasonable to speculate that changes in the brain O_2_ concentrations associated with changes in brain activity or pathological conditions might significantly alter PG production, especially in regions with low O_2_ levels, thus positioning COX-PG as an O_2_ sensor. Together with a well-established vasoactivity of PG (55, 56) and their modulatory effect on neuron activity (57, 58), O_2_-dependent PG regulation might have an important role in rapid brain adaptation to changes in energy and O_2_ demands.

Though not challenged in the present study, other bioactive products of 20:4n6 and other polyunsaturated fatty acids oxidation, including LOX-dependent metabolites and non-enzymatic products of oxidation which have an important role under brain ischemia (53), might be regulated by the same mechanism. This speculation is supported by the similar COX *Km(O*_*2*_*)* values reported for various lipoxygenases that are in the 8–26 μM range (40). Further studies are needed to address this possibility.

In summary, we demonstrated, for the first time, that PG upregulation is highly dependent upon O_2_ availability. The described mechanism for O_2_-dependent PG production which might have a significant role in PG regulation under other pathological or physiological conditions, requires further investigation.

## Data availability

All data are contained in this manuscript.

## Supplemental data

This article contains supplemental data.

## Conflict of interest

The authors declare that they have no conflicts of interest with the contents of this article.
