# Supplementary material for: Exogenous oxygen is required for prostanoid induction under brain ischemia as evidence for a novel regulatory mechanism
Source: J Lipid Res. 2023 Sep 30;64(11):100452. doi: 10.1016/j.jlr.2023.100452 (PMC10630775; doi:10.1016/j.jlr.2023.100452)
Supplement: Supplemental Figure 1 [file mmc1.pdf]

Supplemental Data

Fig. 1S. Confirmation of oxygen probe coordinates.

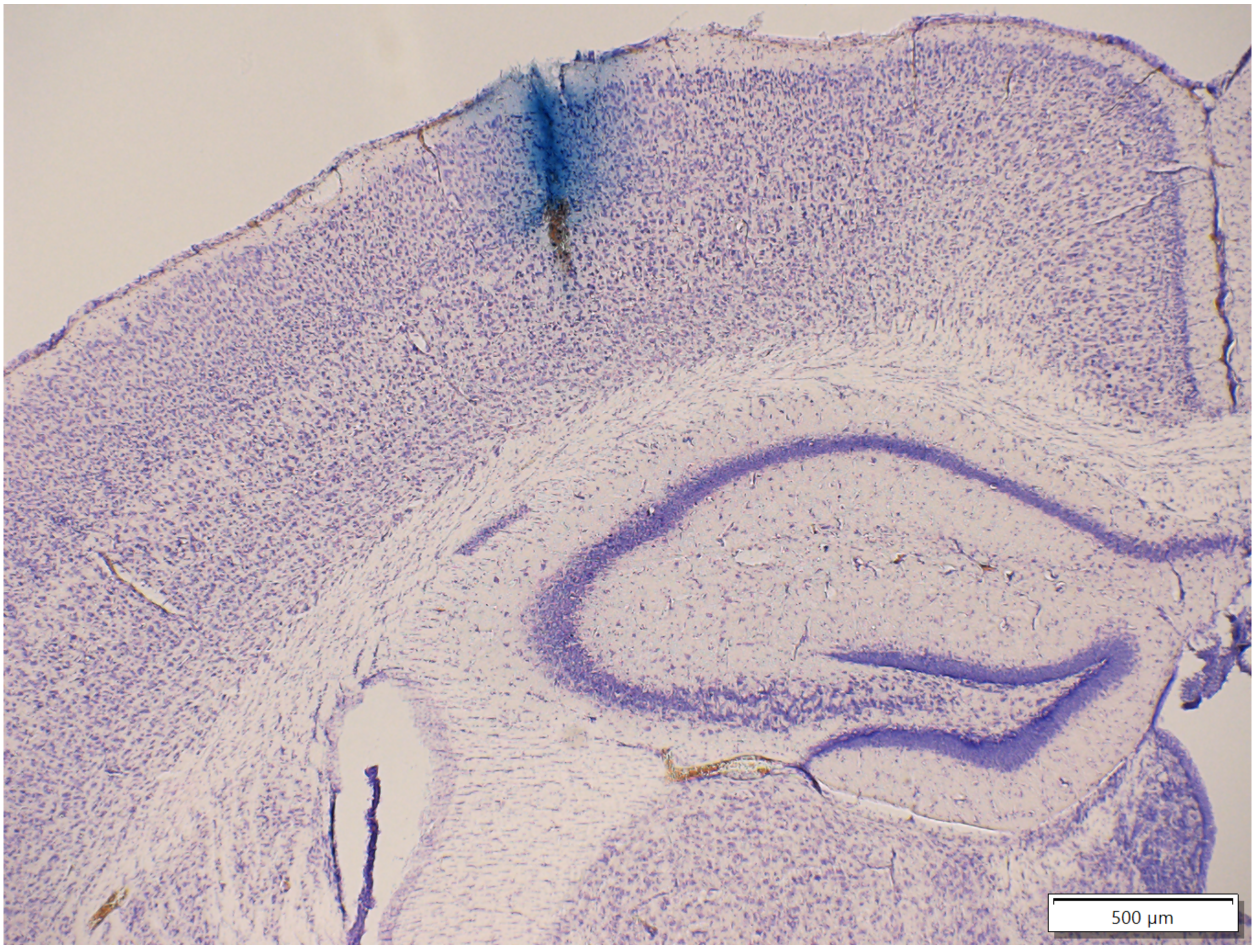

Mice were fixed in a stereotaxic frame followed by a 1 x 1 mm borehole in the cranium. A pulled glass capillary (~100 μm diameter) filled with Evans blue was inserted into the cortex (-1 mm DV, 2 mm ML, and -2 mm AP to bregma). Evans blue dye trace left after capillary removal was visualized in fixed brain slices. A dark stain under dye trace represents fixed red blood cells from trauma caused by a larger,100 μm diameter capillary as opposed to the 10 μm O_2 sensor. For tissue fixation, brain was collected and immersion fixed in 4% paraformaldehyde for 3 days followed by cryopreservation in 30% sucrose for 2 days. Brains were frozen on dry ice and coronally sliced at 40 μm using a Leica CM 3050S cryostat.

Slices were stained with cresyl violet to stain Nissl substance and imaged at 4x using brightfield on an Olympus BX53 light microscope.
